# Exploring the Impact of Serious Games for Cognitive Functions through the Humphrey Fellowship Programme

**DOI:** 10.21315/mjms2018.25.3.1

**Published:** 2018-06-28

**Authors:** Tan Wee Hoe

**Affiliations:** Creative Multimedia Department, Faculty of Art, Computing & Creative Industry, Universiti Pendidikan Sultan Idris, 35900 Tanjong Malim, Perak, Malaysia

**Keywords:** serious games, cognitive, device, medicine, Malaysia

## Abstract

The use of serious games as digital medicine started in recent years as the United States Food and Drug Administration granted Class 1 or 2 device clearance to serious games or game-like technologies. This encouraging trend motivates interdisciplinary cooperation between experts in the medical sciences and the game industry because the Northern American pharmaceutical revenues have exceeded USD1 trillion globally since 2014. The potential of this lucrative business opportunity may attract fund providers and venture capitalists to support game-related research and development projects. The author elaborates his activities and experiences in the United States in FY2017/2018 as a Humphrey fellow from Malaysia. Specifically, the research and development trend of serious games for cognitive function in the academic and the game industry has positive impact on how medical doctors and practitioners in low- and middle-income countries may use or develop games as digital medicine.

The hype created by some brain training games in the United States (U.S.), and their role in improving conditions such as ADHD and Alzheimer’s disease, ended up with a federal crackdown in 2016 as the Federal Trade Commission charged Lumo Labs for overselling the effectiveness and benefits of Lumosity game series (1). Empirical studies began to show evidence that the effects of brain training games may be just placebo; however, some researchers took advantage of the placebo effect as they found out that players’ expectation can actually influence cognitive performance (2). This editorial juxtaposes how a Humphrey fellow from Universiti Pendidikan Sultan Idris (UPSI) explored the impact of games for cognitive function throughout a ten-month Hubert H. Humphrey Fellowship Programme at Pennsylvania State University (PSU) and Yale University, and what professionals in low- and middle-income countries should be mindful of when creating or using games in medical or paramedical contexts (Figure 1).

Explicit use of games as means of teaching and learning at PSU, a member of the Big Ten Academic Alliance (3), is normally limited to individual professors who hold positive perceptions towards games. However, the PSU does offer a course to all faculty and teaching assistants titled Gamification in Online Teaching and Learning (4). A problem facing academic researchers who are interested in conducting research and development (R&D) projects is they have to conceal that they are creating “games” as mobile or interactive technologies in order to secure public funds e.g., the National Science Foundation. Such scenarios may occur in other Big Ten Academic Alliance universities, due to the lack of confidence towards the prospect of game studies. This could also be the case in Malaysia, where games are generally perceived as non-serious entertainment (5), and not a top priority for public R&D fund appropriation. Professors in medical schools who are interested in using games for teaching purposes can package them as “electronic textbooks” or “interactive technologies”. Medical practitioners in hospitals who are interested to initiate or join game-related projects in the U.S. use the terms “software” or “interactive technologies” rather than games to help secure public research funds.

Undergraduate and postgraduate students are constantly encouraged to make games. According to Dr. James Delattre, Assistant Vice President of Research and Director of the Office of Entrepreneurship and Commercialisation, PSU encourages students to work with faculty and community members to develop games or game-like applications. In particular, the Happy Valley LaunchBox was set up to run entrepreneurship programmes, including the Idea TestLab, the FastTrack Accelerator, and the Global Entrepreneurship Week (GEW). In FY2017/2018, two healthcare startups were established: (a) HemoGO, which aims to reduce the necessity of cancer patients to go to a laboratory for regular blood tests (6); and (b) Pathways AI, which enables patients with Parkinson’s disease to track health status and transfer the data to physicians (7). Despite demonstrating convincing features, neither of these startups can commercialise the products before passing the U.S. Food and Drug Administration (FDA) Device Clearance, which is the gold standard for any serious games or game-like apps to be distributed in U.S. Such regulation issues must be taken into consideration if medical professionals or healthcare service providers in developing countries intend to design and develop serious games.

Passing the FDA clearance means physicians in the U.S. can prescribe games as a treatment, which is essential for patients to get insurance reimbursement (8). In other words, serious games can generate revenues in the pharmaceutical industry, which was worth more than USD 300 billion in Northern America in 2017 alone (9). According to Ms Stephanie Wong, the Head of Trade from the Malaysia Digital Economy Corporation Inc. based in Silicon Valley, the market of Malaysia is too small in the eyes of American venture capitalists. This suggests that health professionals in Southeast Asia should consider entering the North American market as Association of Southeast Asian Nations (ASEAN) rather than Malaysia alone (Figure 2). In addition, working under the umbrella of ASEAN may afford R&D teams to conduct product safety and efficacy tests and trials with participants living in different socio-economic conditions across 12 member countries.

The process of creating a game—from an idea, to a prototype game, to a software as medical device (SaMD) that passes FDA clearance—is no doubt a long and expensive journey (8). Therefore, medical researchers in low- and middle-income countries should target for class 1 or 2 with the lowest or low risk-level, as opposed to class 3 or 4 with the highest or serious risk-level set by FDA (10), as suggested by Noah Falstein in the 2018 Game Developers Conference (Figure 3). The success of Akili Interactive Labs in turning NeuroRacer into AKL-T001, a digital health product that passed FDA clearance as a prescription treatment has motivated a lot of serious games partnerships between the medical industry and the game industry (11). NeuroRacer was originally a video game designed as an intervention for top-down modulation deficits in adults aged between 60 to 85 years (12). The game research project was led by Dr Adam Gazzaley, an Associate Professor of neurology, physiology and psychiatry, and the founding director of Neuroscape in University of California, San Francisco (13). The translational nature of neuroscience seems to have afforded experts in medical sciences to work with professionals in other fields to produce marketable innovation—a model which can be replicated in developing countries.

Medical practitioners who are not interested in creating their own games or passing any FDA clearance can still use games to treat patients. In particular, the Lucile Packard Children’s Hospital in Stanford pioneered the use of distraction-based virtual reality (VR) therapy in all patient units, specifically to alleviate pain and anxiety among pediatric patients (14). Since a cardboard VR headset which holds almost any smartphone can cost less than USD5 or RM20 now, the practice of distraction-based VR therapy can be replicated and implemented right away in low-income countries to reduce pain and anxiety.

As the computing technology advances, cybersickness in VR gradually becomes a non-issue (15), which opens up an opportunity to exploit the technology for various pragmatic applications. Within the Yale School of Medicine, the Yale Center for Health and Learning Games houses two labs that investigate serious games: the play2PREVENT Lab and the play4REAL Lab (16) (Figure 4). The play4REAL Lab is developing VR apps that simulate realistic scenarios in school, social activities and at home where targeted players normally experience in real life. The play2PREVENT Lab has produced and evaluated a series of games for preventing tobacco use, substance abuse, HIV and sexually transmitted infections young adolescents. Researchers in the play2PREVENT and play4REAL labs collaborate with game consultants and developers around the world, opening up opportunities for medical professionals and serious game developers from Southeast Asia who are well-versed in serious games.

## Figures and Tables

**Figure 1a:**
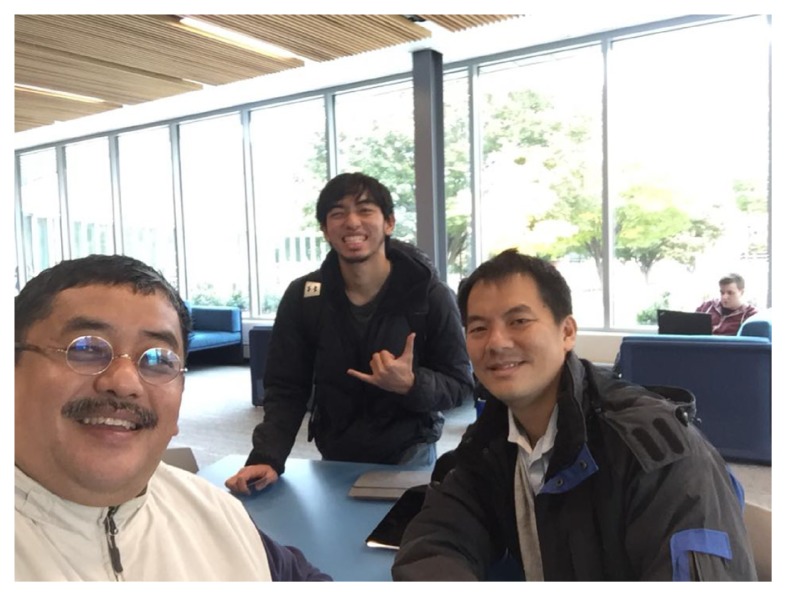
Discussion on the collaboration between UPSI and Universiti Sains Malaysia (USM) in the Global Health Innovation Technology Challenge, the STEM Comic Project and the Gamification of Neuron Man in Pennsylvania State University (PSU) Harrisburg Campus on 10 November 2017. From the left: Professor Dato’ Dr Jafri Malin Abdullah (USM), Mr Adam Hafiz Jafri (PSU), and Associate Professor Dr Tan Wee Hoe (UPSI)

**Figure 1b:**
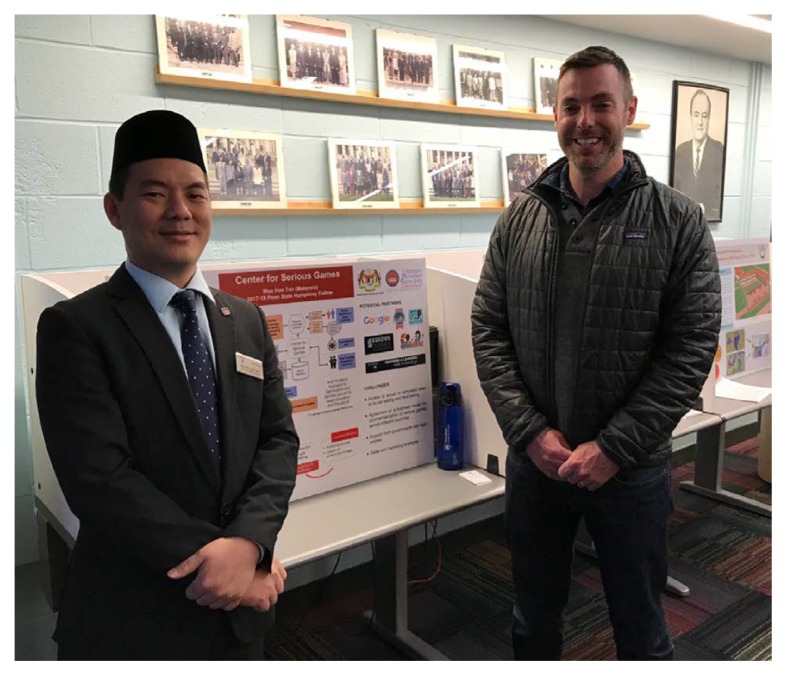
A capstone project of establishing a Center for Serious Games in Malaysia was presented by Associate Professor Dr Tan Wee Hoe (UPSI) at the Office of International Programmes in Penn State College of Education. Assistant Professor Dr Ty Hollett (Penn State College of Education) showed interest in the project

**Figure 1c:**
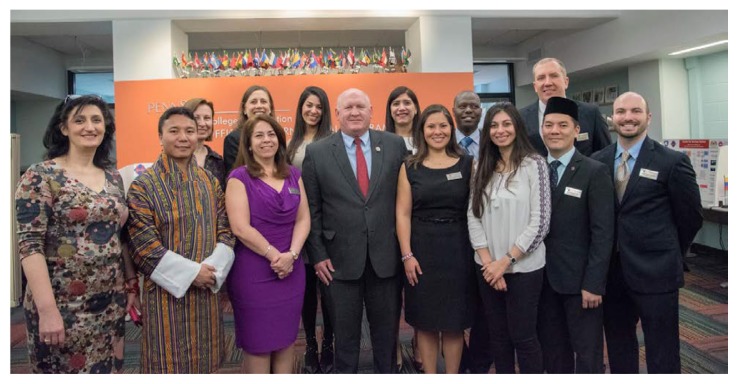
Humphrey Fellows Capstone Project Presentations, on 14 April 2018 at Pennsylvania State University (PSU). From the left: Professor Dr Irma Barbakadze (East European University, Georgia), Mr Kinley Rinchen (Royal University of Bhutan), Dr Natalia Kovalchuk (Ukraine Catholic University), Mdm Viviana del Carmen Gomez-Cuervo (Office of Cuban Airport and Aeronautical Services, Cuba), Assistant Professor Dr Leila Bradaschia (Director of International Programme, PSU), Assistant Professor Dr Hadeel R Bakhsh (Princess Nourah Bint Abdulrahman University, Saudi Arabia), Mr Glenn Thompson (U.S. Representative for Pennsylvania’s 5th District), Ms Ana Carolina Gonzalez Romero (Universidad del Zulia, Venezuela), Ms Maria Rodriguez Arora (La Escuela Superior Politécnica del Litoral, Ecuador), Dr Lazare Rukundwa Sebitereko (President of Eben-Ezer University of Minembwe, Congo), Ms Hoda El Mahdy (Sawiris Foundation for Social Development, Egypt), Mr Sergey Mogilnyy (Seifullin Kazakh Agrotechnical University, Kazakhstan), Associate Professor Dr Tan Wee Hoe (UPSI), and Mr Troy Carl (Outreach Coordinator of Humphrey Fellowship and International Programmes, PSU)

**Figure 2:**
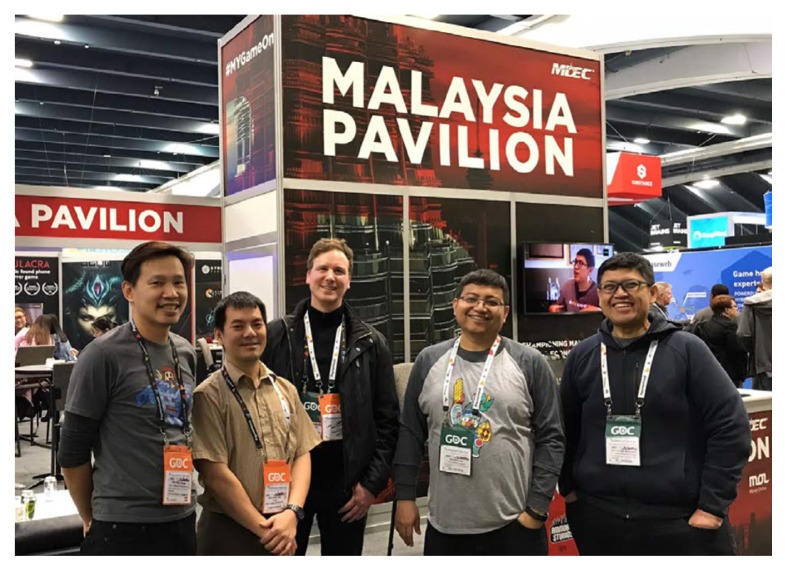
The Malaysia Pavilion booth, set up by the Malaysia Digital Economy Corporation (MDEC) at the Games Developers Conference (21–23 March 2018) in San Francisco. From the left: Mr Siow Aik Wee (MDEC), Associate Professor Dr Tan Wee Hoe (UPSI), Mr Matt Seeney (CEO of Play2Improve, UK), Mr Mohan Low (MDEC), and Mr Hasnul Hadi Samsudin (Vice President of Creative Content & Technologies at MDEC)

**Figure 3:**
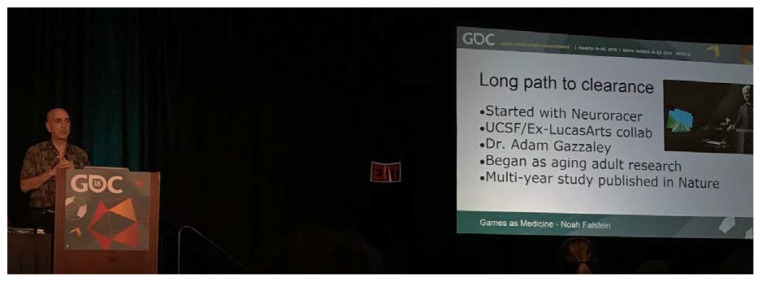
Noah Falstein presented in the 2018 Game Developers Conference on how he worked with game developers to pass the FDA device clearance

**Figure 4a:**
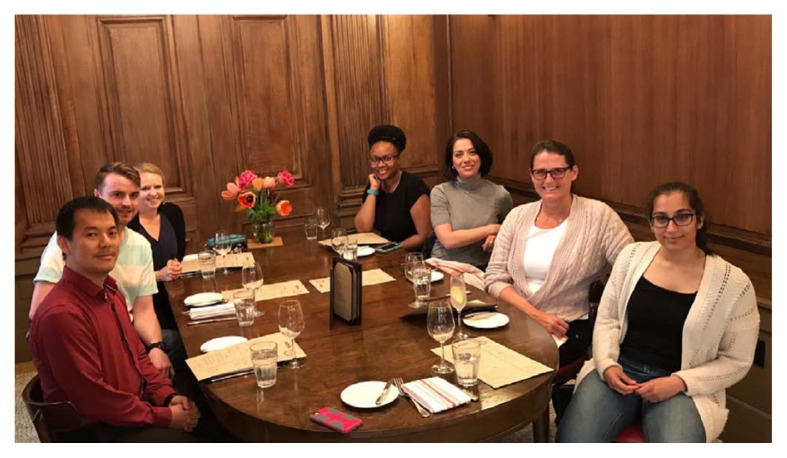
From the left: Associate Professor Dr Tan Wee Hoe (Visiting Research Scientist), Dr Jeffrey Caron (Post-doctoral Associate), Dr Kimberly Hieftje (Deputy Director), Ms Tyra Pendergrass (Associate Director), Dr Claudia-Santi F Fernandes (Post-doctoral Associate), Associate Professor Dr Lynn E. Fiellin (Director) and Ms Trisha Arora (Research Assistant) are members of the Play2PREVENT Lab, in the Yale Center for Health and Learning Games on 8 May 2018

**Figure 4b:**
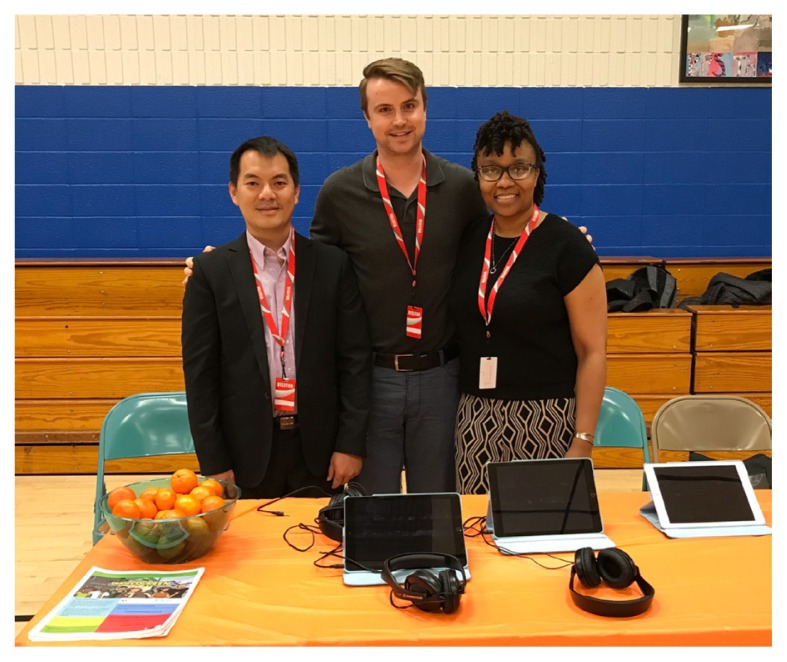
Promoting serious games to students in Peck Place School on 30 April 2018. From the left: Associate Professor Dr Tan Wee Hoe (Visiting Research Scientist), Dr Jeffrey Caron (Post-doctoral Associate), and Ms Tyra Pendergrass (Associate Director)

**Figure 4c:**
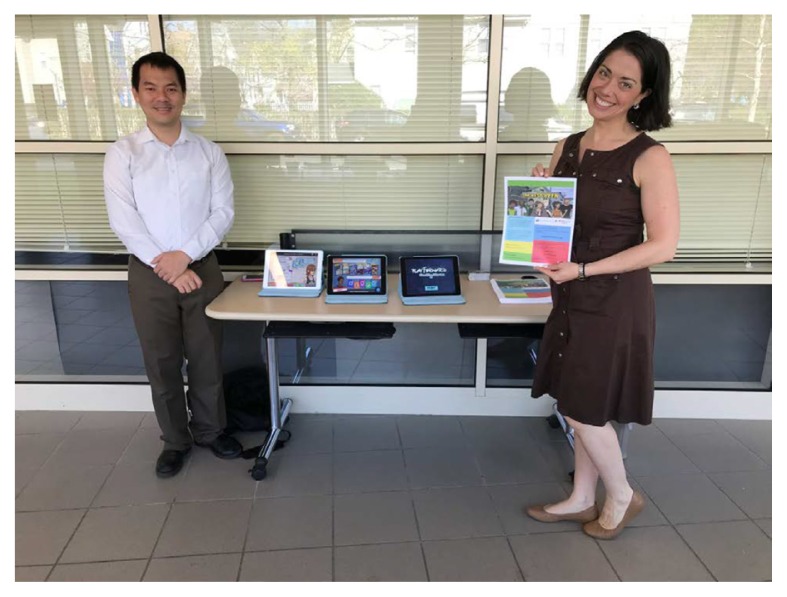
Associate Professor Dr Tan Wee Hoe and Dr Claudia-Santi F Fernandes promoted serious games produced by Play2PREVENT Lab to Yale University community on 3 May 2018

**Figure 4d:**
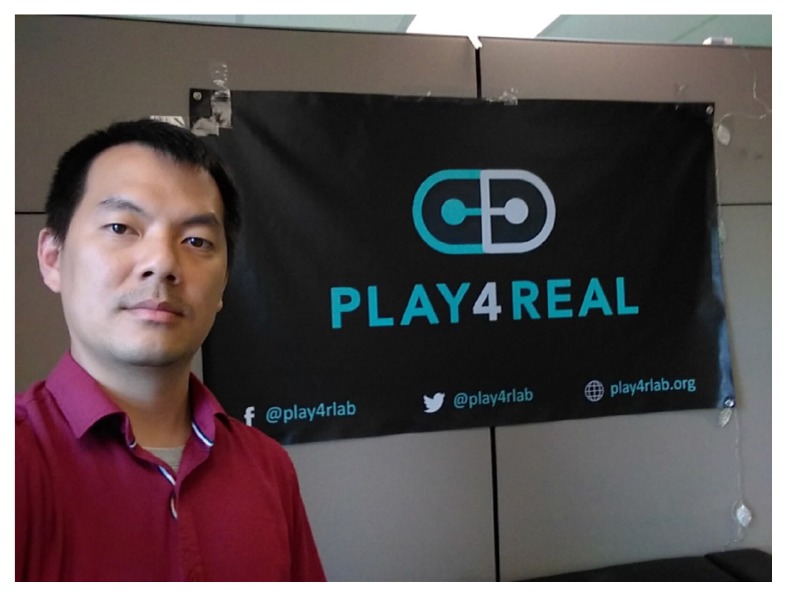
Play4REAL is a lab established under Yale Center for Health and Learning Games to produce VR games

## References

[1] Robbins R (2016). Lumosity reels after federal crackdown on ‘online brain-training’. STAT [Internet].

[2] Robbins R (2016). Mental boost of brain-training games may be just placebo effect. STAT [Internet].

[3] Secor R (2002). Penn State joins the Big Ten and learns to benchmark. New Directions for High Education.

[4] Penn State World Campus (2018). OL3500: Gamification in online teaching and learning.

[5] Tan WH (2015). Gamifikasi dalam pendidikan: pembelajaran berasaskan permainan. Chapter 1, Konsep asas permainan, gamifikasi dan pembelajaran berasaskan permainan.

[6] Toomer L (2018). Penn State students’ app for cancer patients secures first place in IdeaMakers Challenge. The Daily Collegian [Internet].

[7] The Centre County Gazette (2017). Happy Valley Launch Box announces revised accelerator program [Internet].

[8] Falstein N Games as medicine: FDA clearance methods. https://www.gdcvault.com/play/1024956/Games-as-Medicine-FDA-Approval.

[9] Statista (2018). Global pharmaceutical industry–statistics and facts.

[10] Food & Drug Administration (2018). Digital health.

[11] Akili Interactive Labs (2018). Transforming healthcare with therapeutically active digital medicine.

[12] Rusli E (2013). NeuroRacer: a video game to sharpen the mind. Wall Street Journal [Internet].

[13] (2017). Neuroscape Core team.

[14] DeTrempe K (2017). Virtual reality alleviates pain, anxiety for pediatric patients.

[15] Pot-Kolder R, Veling W, Countte J, van der Gaag M (2018). Anxiety partially mediates cybersickness symptoms in immersive virtual reality environments. Cyberpsychology, Behavior, and Social Networking.

[16] Play2PREVENT (2018). Harnessing videogame technology to shape stronger and healthier lives.

